# Integrating Ni(OH)_2_ Nanoparticles on CdS for Efficient Noble-Metal-Free Photocatalytic H_2_ Evolution

**DOI:** 10.3390/molecules29245821

**Published:** 2024-12-10

**Authors:** Zemeng Wang, Piaopiao Wu, Weiya Huang, Kai Yang, Kangqiang Lu, Zhaoguo Hong

**Affiliations:** 1 Jiangxi Provincial Key Laboratory of Functional Crystalline Materials Chemistry, School of Chemistry and Chemical Engineering, Jiangxi University of Science and Technology, Ganzhou 341000, China; mmeng8686@163.com (Z.W.); 19142170804@163.com (P.W.); hweiya@126.com (W.H.); yangkai@jxust.edu.cn (K.Y.); 2 School of Pharmaceutical Sciences, Gannan Medical University, Ganzhou 341000, China

**Keywords:** H_2_ evolution, photocatalysis, cadmium sulfide, nickel hydroxide, cocatalyst

## Abstract

Photocatalytic hydrogen evolution using inexhaustible clean solar energy is considered as a promising strategy. In order to build an efficient photocatalytic hydrogen production system to satisfy the demands of practical applications, it is of great significance to design photocatalysts that offer high activity, low cost, and high stability. Herein, a series of cheap CdS/Ni(OH)_2_ composite photocatalysts were designed and synthesized using the hydrothermal method. The introduction of a Ni(OH)_2_ cocatalyst multiplied the reactive active site of cadmium sulfide and promoted the transfer of photoinduced electrons in a semiconductor. Therefore, CdS/Ni(OH)_2_ composites demonstrate significantly better photocatalytic performance, and the hydrogen production rate of an optimal CdS/5%Ni(OH)_2_ composite is 6.9 times higher than that of blank CdS. Furthermore, the stability test also showed that CdS/Ni(OH)_2_ had good stability. This study aims to serve as a rewarding reference for the development of high-performance composite photocatalysts.

## 1. Introduction

Hydrogen (H_2_) energy is regarded as the cleanest energy source in modern energy systems, as its combustion produces only water. This has drawn significant attention from researchers globally [1,2,3,4]. Conventional H_2_ evolution methods, such as natural gas reforming and coal gasification, rely on fossil fuels and generate carbon dioxide as a by-product. As an alternative to fossil fuels, it is important to develop production methods for hydrogen as a renewable energy fuel [5,6,7,8]. Photocatalytic hydrogen production is universally considered a highly promising approach, in order to build an efficient photocatalytic hydrogen production system to satisfy the demands of practical applications. Thus, designing photocatalysts with inexpensive, high-efficiency and stable feature is essential [9,10,11,12,13]. To achieve a high performance level in solar water splitting, semiconductor photocatalysts must fulfill several requirements, such as having an appropriate band structure, excellent charge conductivity, and strong reactivity. Despite extensive research on metal oxides like TiO_2_, ZnO, and SrTiO_3_ for photocatalytic water reduction reactions, their activity remains insufficient [14,15,16].

Notably, increasing amounts of studies are being conducted on CdS, which demonstrates excellent performance [17]. However, the problem of porous oxidation decomposition (photocorrosion) seriously restrains the capability of CdS [18,19]. In general, loading a proper cocatalyst on the photocatalyst can economically improve the photostability of photocatalysts by wisely consuming or transferring the resulting electron carriers [20,21,22,23]. Therefore, the reasonable introduction of a cocatalyst on CdS photocatalyst is an effective method to reduce CdS photocorrosion and achieve more efficient photocatalytic hydrogen production [24,25]. Studies have shown that cocatalysts can help catalyze multiple reactions at active sites, promote charge separation, prevent the photocorrosion of a CdS-based photocatalyst, and achieve high catalytic performance. Ni(OH)_2_ is a widely used cocatalyst because of its easy availability and abundant active sites [26,27].

In this paper, different proportions of Ni(OH)_2_ cocatalyst are introduced onto CdS with a simple low temperature co-precipitation method through controlling the mass of CdS material and nickel nitrate hexahydrate. Relative to blank CdS, CdS/Ni(OH)_2_ binary hybrid samples show the best H_2_ evolution effect. The H_2_ production rate of CdS/5%Ni(OH)_2_ reaches 10,525.72 μmol/g/h, which is 6.9 times higher than that of blank CdS (1517.91 μmol/g/h). The cyclic experiment result also demonstrates that the introduction of cocatalyst Ni(OH)_2_ can effectively promote the stability of CdS. A series of characterizations validate that a trace amount of Ni(OH)_2_ can offer additional active sites for CdS, thereby effectively improving the separation of photoinduced electron carriers. In addition, incorporating a trace amount of a Ni(OH)_2_ cocatalyst effectively inhibits the photocorrosion of a CdS cube surface, thus enhancing the photocatalytic effect. This study illustrates the significant role of Ni(OH)_2_ as a cocatalyst in photocatalytic hydrogen production and is anticipated to serve as a valuable reference for the further application and evolution of efficient, stable, and cost-effective photocatalysts.

## 2. Results and Discussion

**Figure 1:**
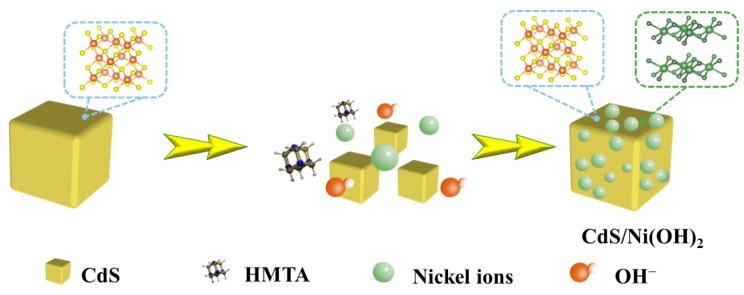
Synthesis diagram of CdS/Ni(OH)_2_ composite photocatalyst.

**Figure 2:**
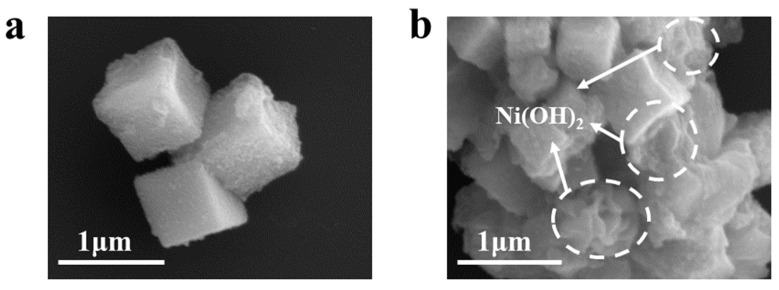
(**a**) SEM diagram of CdS. (**b**) SEM diagram of CdS/Ni(OH)_2_.

The synthesis-process flowchart of CdS/Ni(OH)_2_ composites is displayed in Figure 1. First, CdS-PBA cubes were synthesized by conventional solution reaction at room temperature. Then, Ni(OH)_2_ was introduced into the CdS cube by the one-step hydrothermal method, and the CdS/Ni(OH)_2_ binary composite could be prepared. Since CdS/5%Ni(OH)_2_ composites present optimal photocatalytic performance among the obtained composites, as shown in the hydrogen production activity section, the features of CdS/5%Ni(OH)_2_ composites will be mainly discussed below. Scanning electron microscopy (SEM) can be utilized to study the surface morphology and microstructure of pure CdS and CdS/Ni(OH)_2_ composite photocatalysts. The SEM images of CdS and CdS/Ni(OH)_2_ composites are displayed in Figure 2. After measurement, it was found that the average side length of the CdS cubes is 0.7 micron, while the average diameter of the Ni(OH)_2_ nanoparticles is 35 nanometers. Figure 2a shows that the cadmium sulfide presents a cube structure with a uniform shape, and Figure 2b can clearly validate that Ni(OH)_2_ nanoparticles are tightly loaded onto the CdS surface [28].

**Figure 3:**
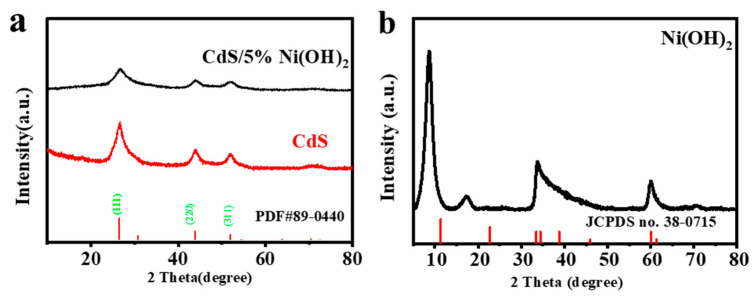
(**a**) XRD pattern of CdS and CdS/Ni(OH)_2_. (**b**) XRD pattern of Ni(OH)_2_.

The X-ray diffraction (XRD) is utilized to determine the phase structure and crystallinity of the synthetic material. As depicted in Figure 3a, blank cadmium sulfide has a sphalerite phase structure. The diffraction peaks at 2θ = 26.4°, 43.8°, and 51.9° can be indexed to the (111), (220), and (311) crystal faces of CdS (PDF#89-0440), respectively. As exhibited in Figure 3b, the diffraction peaks of Ni(OH)_2_ at 8.7°, 17.3°, 33.4°, and 59.9° correspond to the (003), (006), (101), and (110) planes of α-Ni(OH)_2_ (JCPDS no. 38-0715) [29,30]. Notably, no obvious peaks of Ni(OH)_2_ are seen in the spectrum of the CdS/5%Ni(OH)_2_ composite, mainly because the peak intensity of Ni(OH)_2_ is weaker relative to CdS. In addition, the content of Ni(OH)_2_ in the system is rather low, meaning that the peak of Ni(OH)_2_ is obscured by CdS in the XRD pattern.

**Figure 4:**
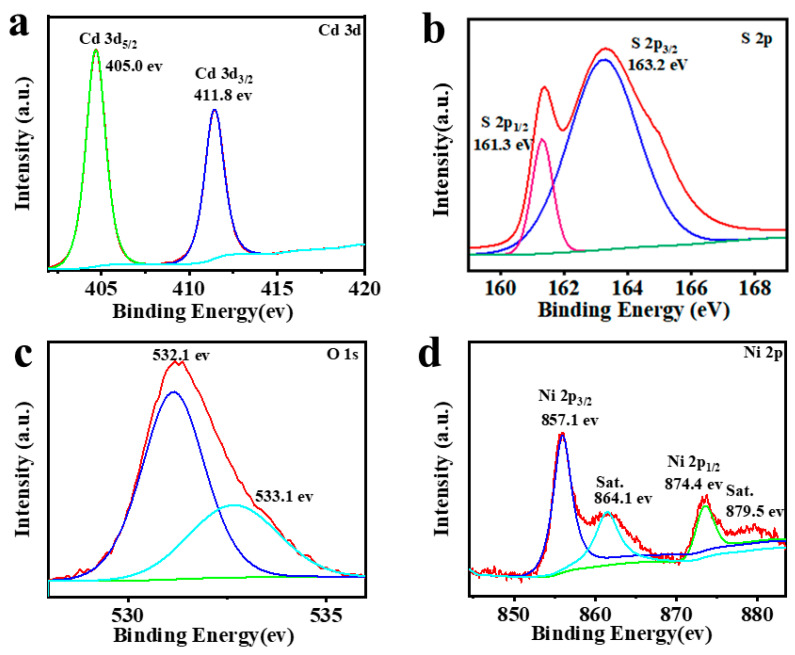
XPS spectra of CdS/Ni(OH)_2_: (**a**) Cd 3d; (**b**) S 2p; (**c**) O 1s; (**d**) Ni 2p.

X-ray photoelectron spectroscopy (XPS) analysis is conducted on the CdS/5%Ni(OH)_2_ composite to further examine its elemental composition and chemical state [31]. As shown in Figure S1, the full spectrum of CdS/Ni(OH)_2_ and peaks in the graph corresponding to S, Cd, O, and Ni can be obviously observed. Figure 4a shows that there are two peaks in the Cd 3d map. The peak at 405.0 eV is associated with the Cd 3d_5/2_, and the peak at 411.8 eV belongs to the Cd 3d_3/2_, indicating the presence of Cd^2+^ in the CdS/5%Ni(OH)_2_ binary composite material [32,33]. Figure 4b depicts the XPS profile of S 2p. It can be seen that the binding energies at 163.2 eV corresponds to S 2p_1/2_ and 161.3 eV belongs to S 2p_3/2_, indicating the presence of S^2−^ in the CdS/5%Ni(OH)_2_ composite [34]. The XPS spectra of O 1s in Figure 4c illustrates that the peak at 532.1 eV belongs to the Ni-OH bond in the composite material, and there is another peak at 533.1 eV due to the H_2_O on the composite material surface [35]. In addition, as presented in Figure 4d, two spin-orbital peaks and two corresponding satellite peaks (labeled as “Sat.”) are exhibited. The peaks attributed to Ni 2p_1/2_ are 874.4 eV, while the peaks belonging to Ni 2p_3/2_ are 857.1 eV. These results indicate that Ni in the form of +2 valence is combined with other elements in the CdS/5%Ni(OH)_2_ composite [26,36]. These XPS results validate that the prepared composite comprises CdS and Ni(OH)_2_, which efficaciously confirms the successful preparation of the CdS/5%Ni(OH)_2_ sample.

**Figure 5:**
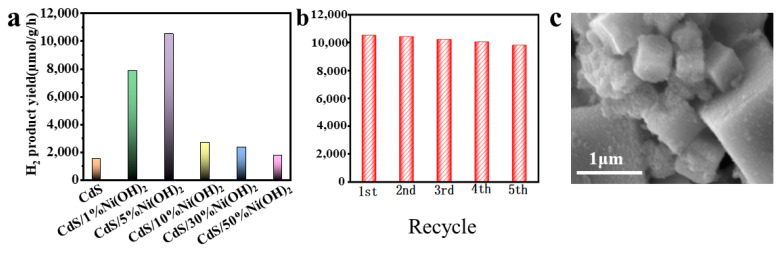
(**a**) Photocatalytic hydrogen production rates of blank CdS and CdS/Ni(OH)_2_ composites. (**b**) Stability plots of the photocatalytic H_2_ production by CdS/5%Ni(OH)_2_. (**c**) SEM image of CdS/5%Ni(OH)_2_ after cyclic test.

To assess the photocatalytic properties of blank CdS and CdS/Ni(OH)_2_ composites with varying amounts of Ni(OH)_2_, photocatalytic hydrogen production is employed. As presented in Figure 5a, the pure CdS shows an inferior photocatalytic performance owing to the high recombination rate of photogenerated electron–hole pairs, leading to the H_2_ evolution rate of only 1517.91 μmol/g/h. After introducing the Ni(OH)_2_ cocatalyst, all CdS/Ni(OH)_2_ composites demonstrate improved H_2_ evolution performance relative to the blank CdS. As the content of Ni(OH)_2_ increases, the hydrogen yield gradually rises. Significantly, the optimal H_2_ evolution rate (10,525.72 μmol/g/h) is shown by the CdS/5%Ni(OH)_2_ composite, which is approximately six times higher than that of CdS alone. Nevertheless, the H_2_ evolution activity decreases when the Ni(OH)_2_ content increases continuously. The photocatalytic performance of CdS/50%Ni(OH)_2_ is almost equivalent to that of the blank CdS. This may due to the remarkable shielding effect of Ni(OH)_2_, which reduces a certain amount of photocatalytic active sites and diminishes the CdS cube’s ability to absorb visible light [37]. Table 1 demonstrates that the photocatalytic hydrogen production rate of the CdS/Ni(OH)_2_ composite material exceeds that of similar photocatalysts previously reported in the literature. Since CdS is transition metal sulfide, it has been reported that CdS is likely to suffer severe photocorrosion [18,19,24,38], resulting in poor stability and a short usable life, which seriously impede the long-term development of the catalyst and the realization of industrial applications. As exhibited in Figure 5b, no remarkable deactivation has been observed in the CdS/5%Ni(OH)_2_ composite after five cycles, indicating the outstanding stability of the CdS/5%Ni(OH)_2_ composite. The SEM image of the CdS/5%Ni(OH)_2_ composite after the cyclic test (Figure 5c) shows that the morphology of the composite remains unchanged, further confirming the relatively great stability of the composite.

**Figure 6:**
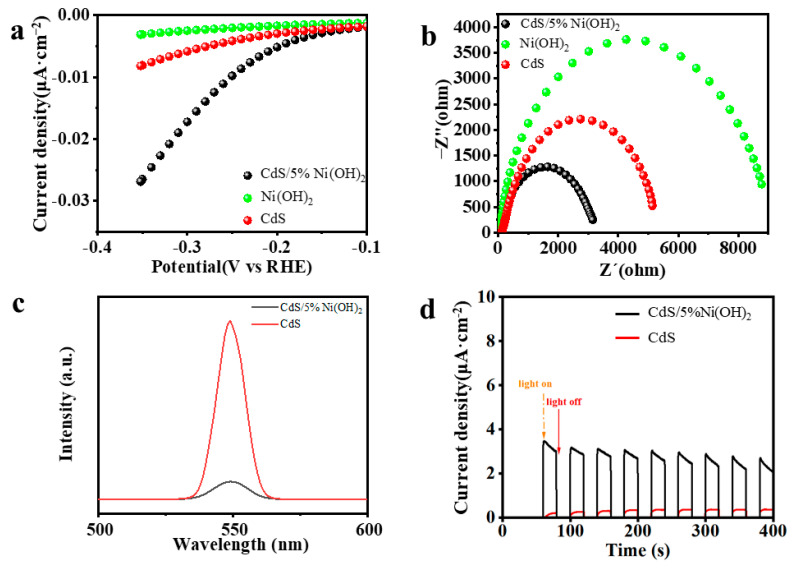
(**a**) Polarization curves. (**b**) EIS Nyquist plots. (**c**) Steady-state photoluminescence (PL) emission spectra. (**d**) Transient photocurrent spectra.

Photo/electrochemical tests are employed to further assess the efficiency of photogenerated carrier transfer, the electrical conductivity, and the active area of obtained samples, in order to investigate the factors resulting in the superior photocatalytic performance of CdS/5%Ni(OH)_2_ relative to pure CdS [48]. To evaluate the H_2_ evolution performance of CdS, Ni(OH)_2_ and CdS/5%Ni(OH)_2_ samples, linear sweep voltammetry (LSV) is initially employed. Figure 6a depicts the polarization curve of CdS, Ni(OH)_2_, and CdS/5%Ni(OH)_2_ composites. It can be obviously seen that the overpotential of CdS/5%Ni(OH)_2_ is less than single Ni(OH)_2_ and blank CdS at the same current density. This illustrates that the reduction capacity of the CdS/5%Ni(OH)_2_ binary composite sample is the strongest, and the hydrogen production effect is the best, which is in accordance with the data of catalyst hydrogen production activity, and in line with the expected regulation [49]. Electrochemical impedance spectroscopy (EIS), steady-state photoluminescence (PL) spectra, and the instantaneous photocurrent (IT) of pure CdS and CdS/5%Ni(OH)_2_ are utilized to investigate the charge separation and transfer of the obtained samples. As depicted in Figure 6b, the curvature radius of the impedance of single Ni(OH)_2_ is the largest among the three samples, indicating its poor electrical conductivity [50]. However, it is worth noting that after including Ni(OH)_2_, the curvature radius of the impedance diagram of CdS/5%Ni(OH)_2_ composite material is not immensely increased relative to that of blank CdS. On the contrary, there is a certain degree of decrease in the curvature radius of CdS/5%Ni(OH)_2_, which indicates that adding a trace amount of Ni(OH)_2_ improves the transfer rate of electrons, and does not simultaneously reduce the conductivity of the composite material [51]. As presented in Figure 6c, the steady-state photoluminescence (PL) spectra show that the PL intensity of the CdS/5%Ni(OH)_2_ composite sample is much lower than that of blank CdS. Generally, stronger fluorescence intensity indicates a more severe recombination of electron–hole pairs’ material. Therefore, the results indicate that the introduction of the cocatalyst Ni(OH)_2_ efficaciously suppresses the recombination of photogenerated carriers of CdS/5%Ni(OH)_2_ [52]. As depicted in Figure 6d, it can be apparently speculated from the low optical current density of CdS that the photogenerated carrier separation efficiency of CdS is poor. Nevertheless, the optical current density of the CdS/5%Ni(OH)_2_ composite is significantly improved after the addition of Ni(OH)_2_, relative to that of pure CdS, indicating that the CdS/5%Ni(OH)_2_ composite exhibits an enhanced separation efficiency of both the hole (h^+^) and the electron (e^−^) [53]. Above all, the outcomes of these photo/electrochemical tests confirm that the introduction of Ni(OH)_2_ cocatalyst can boost the transfer of the photogenerated charges carried in CdS/5%Ni(OH)_2_, resulting in the improved performance of the photocatalytic H_2_ evolution of CdS/5%Ni(OH)_2_. The cyclic voltammetry (CV) curve of the catalyst can be used to measure the reactive area.

**Figure 7:**
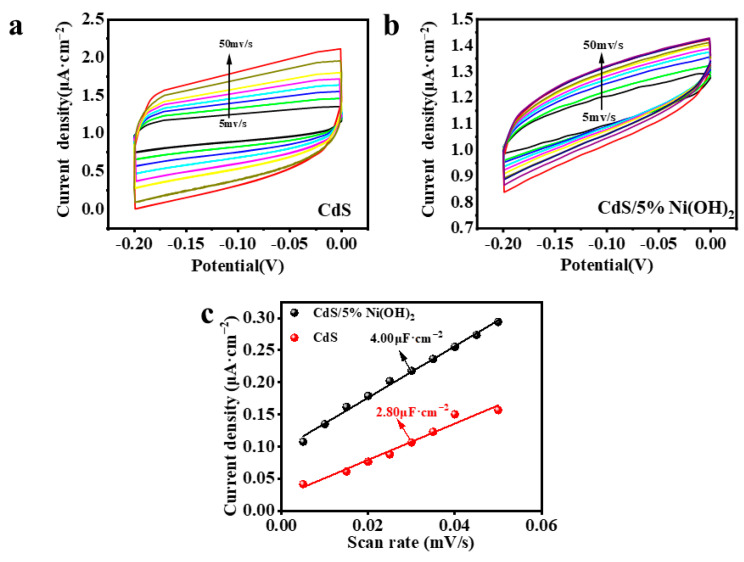
Cyclic voltammetry curves of (**a**) CdS and (**b**) CdS/Ni(OH)_2_. (**c**) Current density-scan rate plots for CdS and CdS/Ni(OH)_2_.

Obviously, the relative surface area of the CdS/5%Ni(OH)_2_ composite is superior to that of pure CdS (Table S1), indicating that the introduction of the Ni(OH)_2_ cocatalyst enhances the surface area of the composite. The increased specific surface area of the CdS/5%Ni(OH)_2_ composite suggests a higher number of exposed reactive sites, which in turn contributes to the improved photocatalytic performance of the sample. Also, the information regarding the chemical reaction area of the pure CdS can be seen in Figure 7a,b, indicated by the cyclic voltammetry test on the CdS/5%Ni(OH)_2_ composite. As presented in Figure 7c, the double-layer capacitance of the CdS/5%Ni(OH)_2_ composite (4.00 µF·cm^−2^) is substantially greater than CdS (2.80 µF·cm^−2^), which robustly validates that the CdS/5%Ni(OH)_2_ composite has a higher amount of active sites compared to CdS.

**Figure 8:**
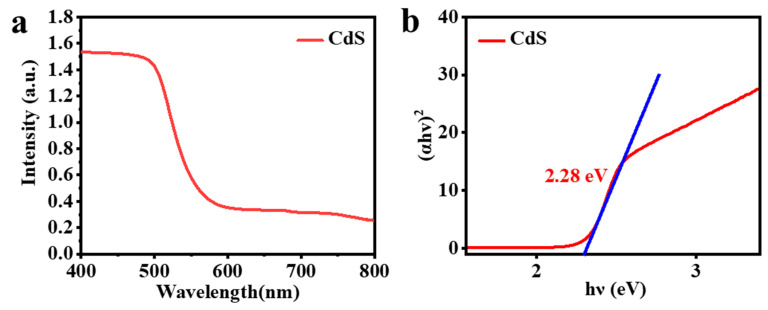
(**a**) UV–vis diffuse reflectance spectra (DRS) of CdS. (**b**) The band-gap energy of CdS.

**Figure 9:**
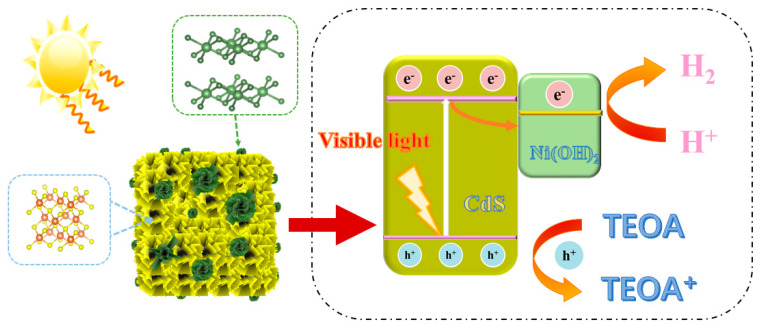
Mechanism of CdS/Ni(OH)_2_ photocatalytic hydrogen evolution in TEOA.

The band-structure information of CdS is obtained using the UV–vis absorption spectra displayed in Figure 8a. The Tauc equation, (αhν)^2^ = A(hν − Eg), is employed to determine the band-gap energy (Eg) of the synthesized samples, where α represents the absorption coefficient, ν is the frequency of light, h is Planck’s constant, and A is the proportionality constant. As exhibited in Figure 8b, it can be seen that the Eg of CdS is 2.28 eV, which indicates that CdS has a good visible-light absorption capacity. Taking into account the previous dialogue, the following mechanism of photocatalytic hydrogen production toward CdS/Ni(OH)_2_ is put forward. As depicted in Figure 9, electrons in CdS are excited after the absorption of visible light and subsequently migrate to the conduction band of CdS, while holes in the CdS valence band are produced. The photoexcited electrons then move to the Ni(OH)_2_ site. Protons from water combine with these photogenerated electrons to generate H_2_. In addition, TEOA is oxidized by previously accumulating photoinduced holes in the valence band of CdS, resulting in the production of oxide triethanolamine. Consequently, the introduction of a small amount of Ni(OH)_2_ greatly enhances the efficient separation of electron–hole pairs and provides abundant active sites, therefore immensely promoting the photocatalytic H_2_ production of CdS/5%Ni(OH)_2_ composites.

## 3. Experimental

### 3.1. Chemicals and Materials

Potassium permanganate (KMnO_4_), 30% hydrogen peroxide (H_2_O_2_), concentrated sulfuric acid (H_2_SO_4_), concentrated hydrochloric acid (HCl), triethanolamine ((HOCH_2_CH_2_)_3_N), anhydrous ethanol (C_2_H_5_OH), nickel nitrate (Ni(NO_3_)_3_·6H_2_O), N,N-dimethylformamide (HCON(CH_3_)_2_), sodium citrate (C_6_H_5_O_7_Na_3_), hexamethyltetramine (C_6_H_12_N_4_), potassium cobalt cyanide (K_3_[Co(CN)]_6_), cadmium acetate dihydrate (C_2_H_7_CdO_4_), and sodium sulfide (Na_2_S·9H_2_O) were used.

### 3.2. Preparation of CdS Cubes

Referring to previous studies, CdS cubes need to be prepared from Cd-based Prussian blue analogs (Cd-PBA) with hollow and framework structures [54]. Briefly, Cd(Ac)_2_·2H_2_O (0.1383 g), polyvinylpyrrolidone (PVP) (1.00 g), and C_6_H_5_Na_3_O_7_ (0.10 g) were introduced into a beaker with 30 mL of deionized water. Subsequently, the K_3_[(Co(CN)_6_] (0.13 g) was dissolved into a separate beaker containing 30 mL of deionized water and then blended with the previously prepared solution. K_3_[Co(CN)_6_] was used to react with the CdS-based metal precursor to generate Cd-PBA. After 30 min of magnetic stirring, the solution was aged for 1 h, centrifuged to collect the outcome, and washed with ethanol 3 times to obtain the Cd-PBA cube solution. Then, the prepared solution was added to 20 mL aqueous solution containing 100 mmol Na_2_S and continuously stirred. After a 2 h reaction, the products were then collected using centrifugation. Eventually, by washing the products three times with ethanol, and drying them in a vacuum at 333 K for 12 h, an aurantium crystalline was obtained.

### 3.3. Synthesis of CdS/Ni(OH)_2_ Binary Composite

In a typical experiment, CdS cube material (0.14 g), a certain amount of nickel nitrate hexahydrate and 10 mL deionized water were mixed. The two solutions were combined and stirred for 30 min. Afterwards, Ni(NO_3_)_3_·6H_2_O (0.06451 g) and hexamethylenetetramine (HMTA) (0.3505 g) were dissolved in the above solution. Subsequently, the resulting solution was heated to 363 K and stirred adequately. After the solution was cooled to room temperature, it was centrifuged and the samples were collected and rinsed three times with deionized water. The CdS/Ni(OH)_2_ composites were obtained through ultimate vacuum drying at 333 K for 12 h. By controlling the mass ratio of nickel nitrate hexahydrate, the quality score of Ni(OH)_2_ in the composite material can be adjusted by 1%, 5%, 10%, 30% and 50%, respectively.

### 3.4. Evaluation of the Photocatalytic H_2_ Evolution Performance 

The photocatalytic H_2_ production was carried out in a 50 mL sealed quartz reactor. A total of 15 mg of sample, 5 mL of ionized water as a proton donor, and 1 mL of triethanolamine as a sacrifice agent were put in the quartz reactor and sonicated in the ultrasonic machine for 3 min to ensure the solution was completely mixed. After sealing, high-purity argon was introduced, and the mixture was stirred for 30 min to remove air and fill the quartz reactor with protective gas. As the light source, a 300 W xenon lamp (λ ≥ 420 nm) with a simulated light intensity of 311.8 mW/cm^2^ was picked. After irradiation for 2 h, 1 mL of gas was extracted for gas chromatography analysis to determine the hydrogen yield in the reaction. The product was then analyzed by chromatography. The hydrogen peak area was recorded, and the argon peak area was used to verify whether the experiment exhibited gas leakage. Additionally, a cycle experiment was conducted to test its stability. The waste liquid in the quartz reactor was centrifuged. The centrifuged solid was then washed three times with absolute ethanol. Afterwards, the resulting solid was vacuum-dried at 333 K for 12 h. After sample drying, the hydrogen production experiment was repeated five times.

### 3.5. Characterization Methods

The crystal phase structure and composition of the catalyst were determined by X-ray diffraction (Bruker D8 Advance, Bruker Corporation, Saarbrucken, Germany). A scanning electron microscope (FESEM Zeiss Sigma 500, Zeiss, Oberkochen, Germany) was used to analyze the morphology and microstructure of the composite photocatalyst. The composition and valence of the composite photocatalyst were analyzed by Thermo Fisher K-Alpha Plus (X-ray photoelectron spectroscopy) (Thermo Fisher, Waltham, MA, USA). In H_2_ evolution performance evaluation, the 300 W xenon lamp (PLS-SXE300D, Perfectlight, Beijing, China) and the gas chromatograph (GC7900, Techcomp, Shanghai, China) were employed. Photoluminescence (PL) spectra were obtained using a spectrofluorometer (FLS 980, Edinburgh Instruments Ltd., Edinburgh, UK) with an excitation wavelength of 500 nm. Furthermore, all the electrochemical measurements of the photocurrent and the electrochemical impedance spectra (EIS) were carried out in the three-electrode cell, in which Ag/AgCl was used as a reference electrode, a Pt wire was used as a counter electrode, and an indium in oxide (ITO) conductive glass was used with the samples as a working electrode in 0.1 M Na_2_SO_4_ electrolyte (pH = 7.56); all measurements were carried out on CH Instruments’ CHI-660E electrochemical workstation (Shanghai Chenhua CHI-660E, Shanghai, China). The specific surface area and pore size of the composite photocatalyst were determined by nitrogen physical adsorption desorption (ASAP2020). The UV–visible diffuse reflectance spectrometer (DRS, Shimadzu UV-2600, Kyoto, Japan) was utilized to test the optical response of the catalyst.

## 4. Conclusions

In summary, using a simple hydrothermal method, the CdS/Ni(OH)_2_ composites are synthesized. Compared with blank CdS and blank Ni(OH)_2_, binary CdS/Ni(OH)_2_ presents superior performance, and the hydrogen production rate of the optimal CdS/5%Ni(OH)_2_ achieved 10,525.72 μmol/g/h; it can be seen from the hydrogen evolution activity that the co-catalyst load is a crucial factor. The series of characterizations confirms that a trace amount of Ni(OH)_2_ cocatalyst can offer an increased amount of active sites for H_2_ production, and effectively boost the separation of photogenerated charge carriers, thus improving the photocatalytic effect. This study is anticipated to serve as a practical reference for the conscious design of coupled photocatalysts aimed at hydrogen production and the further evolution of selective high-performance composite photocatalysts.

## Figures and Tables

**Table 1:** Hydrogen production performance of various CdS-based photocatalysts.

Photocatalysts	Sacrificial Agents	H_2_ (μmol∙g^−1^∙h^−1^)	Reference
CdS/5%Ni(OH)_2_	TEOA	10,525.72	this work
Ni@NiO/CdS	TEOA	4380	[39]
CdS/TiO_2_@Ti_3_C_2_	TEOA	3115	[40]
AgBr/CdS	TEOA	5406	[41]
CdS@MoS_2_	benzyl alcohol	9033	[42]
CdS/MIL-53 (Fe)	benzyl alcohol	2334	[43]
Co/CdS NRs	benzyl alcohol	1302.0	[44]
Ni/ZnCdS	benzyl alcohol	5753.5	[45]
CuS/CdS	lactic acid (10 vol%)	5617	[46]
ZnO-Cu-CdS	Glycerol	4655	[47]

## Data Availability

The original contributions presented in the study are included in the article/Supplementary Material, further inquiries can be directed to the corresponding authors.

## Supplementary Materials

The following supporting information can be downloaded at: https://www.mdpi.com/article/10.3390/molecules29245821/s1, Figure S1. XPS spectra for the survey spectra of the CdS/5%Ni(OH)_2_ composite. Table S1 Surface Areas of Samples.

## References

[1] Zheng M., Wu P., Li L., Yu F., Ma J. (2023). Adsorption/desorption behavior of ciprofloxacin on aged biodegradable plastic PLA under different exposure conditions. J. Environ. Chem. Eng..

[2] Wang J.-H., Yang S.-W., Ma F.-B., Zhao Y.-K., Zhao S.-N., Xiong Z.-Y., Cai D., Shen H.-D., Zhu K., Zhang Q.-Y. (2023). RuCo alloy nanoparticles embedded within N-doped porous two-dimensional carbon nanosheets: A high-performance hydrogen evolution reaction catalyst. Tungsten.

[3] Yang Y., Wu J.S., Cheng B., Zhang L.Y., Al-Ghamdi A.A., Wageh S., Li Y.J. (2022). Enhanced photocatalytic H_2_-production activity of CdS nanoflower using single atom Pt and graphene quantum dot as dual cocatalysts. Chin. J. Struct. Chem..

[4] Augustyn V., Simon P., Dunn B. (2014). Pseudocapacitive oxide materials for high-rate electrochemical energy storage. Energy Environ. Sci..

[5] Zheng X.L., Yang Y.Q., Song Y.M., Ma Z.X., Gao Q.Z., Liu Y.H., Li J., Wu X., Wang X.B., Mao W.H. (2023). Recent advances in photocatalytic hydrogen evolution of AgIn_5_S_8_-based photocatalysts. Interdiscip. Mater..

[6] Jiang J., Xiong Z., Wang H., Liao G., Bai S., Zou J., Wu P., Zhang P., Li X. (2022). Sulfur-doped g-C_3_N_4_/g-C_3_N_4_ isotype step-scheme heterojunction for photocatalytic H_2_ evolution. J. Mater. Sci. Technol..

[7] Li M., Van Der Veer M., Yang X., Weng B., Shen L., Huang H., Dong X., Wang G., Roeffaers M.B.J., Yang M.-Q. (2024). Twin boundary defect engineering in Au cocatalyst to promote alcohol splitting for coproduction of H_2_ and fine chemicals. J. Colloid Interface Sci..

[8] Chang X.X., Wang T., Gong J.L. (2016). CO_2_ photo-reduction: Insights into CO_2_ activation and reaction on surfaces of photocatalysts. Energy Environ. Sci..

[9] Wu Y.-H., Yan Y.-Q., Wei Y., Wang J., Li A., Huang W.-Y., Zhang J.-L., Yang K., Lu K.-Q. (2024). Decorating ZnIn_2_S_4_ with earth-abundant Co_9_S_8_ and Ni_2_P dual cocatalysts for boosting photocatalytic hydrogen evolution. Int. J. Hydrogen Energy.

[10] Su B., Zheng M., Lin W., Lu X.F., Luan D., Wang S., Lou X.W. (2023). S-scheme Co_9_S_8_@Cd_0.8_Zn_0.2_S-DETA hierarchical nanocages bearing organic CO_2_ activators for photocatalytic syngas production. Adv. Energy Mater..

[11] Ma M.-Y., Yu H.-Z., Deng L.-M., Wang L.-Q., Liu S.-Y., Pan H., Ren J.-W., Maximov M.Y., Hu F., Peng S.-J. (2023). Interfacial engineering of heterostructured carbon-supported molybdenum cobalt sulfides for efficient overall water splitting. Tungsten.

[12] Gao J.-X., Tian W.-J., Zhang H.-Y. (2022). Progress of Nb-containing catalysts for carbon dioxide reduction: A minireview. Tungsten.

[13] Chen S.S., Qi Y., Li C., Domen K., Zhang F.X. (2018). Surface Strategies for Particulate Photocatalysts toward Artificial Photosynthesis. Joule.

[14] Pan Y., Liang W.Z., Wang Z.P., Gong J.J., Wang Y.C., Xu A.J., Teng Z.Y., Shen S.J., Gu L., Zhong W.W. (2024). Facile synthesis of Pt clusters decorated TiO_2_ nanoparticles for efficient photocatalytic degradation of antibiotics. Interdiscip. Mater..

[15] Xu Y.S., Liang Y.H., He Q.Q., Xu R.L., Chen D.C., Xu X.J., Hu H.W. (2022). Review of doping SrTiO_3_ for photocatalytic applications. Bull. Mater. Sci..

[16] Pérez-Larios A., Torres-Ramos I., Zanella R., Rico J.L. (2022). Ti-Co mixed oxide as photocatalysts in the generation of hydrogen from water. Int. J. Chem. React. Eng..

[17] Gao R.Q., He H., Bai J.X., Hao L., Shen R.C., Zhang P., Li Y.J., Li X. (2022). Pyrene-benzothiadiazole-based polymer/CdS 2D/2D organic/inorganic hybrid S-scheme heterojunction for efficient photocatalytic H_2_ evolution. Chin. J. Struct. Chem..

[18] Ke D.N., Liu S.L., Dai K., Zhou J.P., Zhang L., Peng T.Y. (2009). CdS/regenerated cellulose nanocomposite films for highly efficient photocatalytic H_2_ production under visible light irradiation. J. Phys. Chem. C.

[19] Kudo A., Miseki Y. (2009). Heterogeneous photocatalyst materials for water splitting. Chem. Soc. Rev..

[20] Weng Z., Lin Y., Han B., Zhang X., Guo Q., Luo Y., Ou X., Zhou Y., Jiang J. (2023). Donor-acceptor engineered g-C_3_N_4_ enabling peroxymonosulfate photocatalytic conversion to ^1^O_2_ with nearly 100% selectivity. J. Hazard. Mater..

[21] Zou J., Wu S., Liu Y., Sun Y., Cao Y., Hsu J.-P., Shen Wee A.T., Jiang J. (2018). An ultra-sensitive electrochemical sensor based on 2D g-C_3_N_4_/CuO nanocomposites for dopamine detection. Carbon.

[22] Li S., Dong K., Cai M., Li X., Chen X. (2024). A plasmonic S-scheme Au/MIL-101(Fe)/BiOBr photocatalyst for efficient synchronous decontamination of Cr(VI) and norfloxacin antibiotic. eScience.

[23] Yang F., Hu P., Yang F., Hua X.-J., Chen B., Gao L., Wang K.-S. (2023). Photocatalytic applications and modification methods of two-dimensional nanomaterials: A review. Tungsten.

[24] Hu Y., Gao X.H., Yu L., Wang Y.R., Ning J.Q., Xu S.J., Lou X.W. (2013). Carbon-Coated CdS Petalous Nanostructures with Enhanced Photostability and Photocatalytic Activity. Angew. Chem. Int. Edit..

[25] Liu Z.-Y., Lin Y.-D., Hao Y., Chen H.-N., Guo Z.-W., Li X.-X., Zheng S.-T. (2022). Recent advances in polyoxoniobate-catalyzed reactions. Tungsten.

[26] Lu K.-Q., Li Y.-H., Zhang F., Qi M.-Y., Chen X., Tang Z.-R., Yamada Y.M.A., Anpo M., Conte M., Xu Y.-J. (2020). Rationally designed transition metal hydroxide nanosheet arrays on graphene for artificial CO_2_ reduction. Nat. Commun..

[27] Qin Z.X., Chen Y.B., Wang X.X., Guo X., Guo L.J. (2016). Intergrowth of Cocatalysts with Host Photocatalysts for Improved Solar-to-Hydrogen Conversion. ACS Appl. Mater. Interfaces.

[28] Zhang W., Wang Y.B., Wang Z., Zhong Z.Y., Xu R. (2010). Highly efficient and noble metal-free NiS/CdS photocatalysts for H_2_ evolution from lactic acid sacrificial solution under visible light. Chem. Commun..

[29] Nagaraju G., Cha S.M., Yu J.S. (2017). Ultrathin nickel hydroxide nanosheet arrays grafted biomass-derived honeycomblike porous carbon with improved electrochemical performance as a supercapacitive material. Sci. Rep..

[30] Jia D.D., Gao H.Y., Dong W.J., Fan S., Dang R., Wang G. (2017). Hierarchical α-Ni(OH)_2_ Composed of Ultrathin Nanosheets with Controlled Interlayer Distances and Their Enhanced Catalytic Performance. ACS Appl. Mater. Interfaces.

[31] Pérez-Larios A., Rico J.L., Anaya-Esparza L.M., Vargas O.A.G., González-Silva N., Gómez R. (2019). Hydrogen Production from Aqueous Methanol Solutions Using Ti–Zr Mixed Oxides as Photocatalysts under UV Irradiation. Catalysts.

[32] Seo O.R., Azizar G.A.B., Hong J.W. (2024). Multi-synergies of hollow CdS cubes on MoS_2_ sheets for enhanced visible-light-driven photocatalysis. Appl. Surf. Sci..

[33] Xie Y.P., Zheng Y., Yang Y., Jiang R., Wang G., Zhang Y., Zhang E., Zhao L., Duan C.-Y. (2018). Two-dimensional nickel hydroxide/sulfides nanosheet as an efficient cocatalyst for photocatalytic H_2_ evolution over CdS nanospheres. J. Colloid Interface Sci..

[34] Zhang H., Gao Y., Meng S., Wang Z., Wang P., Wang Z., Qiu C., Chen S., Weng B., Zheng Y.M. (2024). Metal Sulfide S-Scheme Homojunction for Photocatalytic Selective Phenylcarbinol Oxidation. Adv. Sci..

[35] Weng Z., Lin Y., Guo S., Zhang X., Guo Q., Luo Y., Ou X., Ma J., Zhou Y., Jiang J. (2023). Site Engineering of Covalent Organic Frameworks for Regulating Peroxymonosulfate Activation to Generate Singlet Oxygen with 100 % Selectivity. Angew. Chem. Int. Ed..

[36] Lu K.-Q., Hao J.-G., Wei Y., Weng B., Ge S., Yang K., Lu S., Yang M.-Q., Liao Y. (2023). Photocatalytic conversion of diluted CO_2_ into tunable syngas via modulating transition metal hydroxides. Inorg. Chem..

[37] Jiang Q.Q., Sun L., Bi J.H., Liang S.J., Li L.Y., Yu Y., Wu L. (2018). MoS_2_ Quantum Dots-Modified Covalent Triazine-Based Frameworks for Enhanced Photocatalytic Hydrogen Evolution. ChemSusChem.

[38] Jing D.W., Guo L.J. (2006). A novel method for the preparation of a highly stable and active CdS photocatalyst with a special surface nanostructure. J. Phys. Chem. B.

[39] Zhang L.J., Zhu X.F., Zhao Y.Y., Zhang P.Y., Chen J., Jiang J.L., Xie T.F. (2019). The photogenerated charge characteristics in Ni@NiO/CdS hybrids for increased photocatalytic H_2_ generation. RSC Adv..

[40] Meeran M.N., Haridharan N., Shkir M., Algarni H., Reddy V.R.M. (2022). Rationally designed 1D CdS/TiO_2_@Ti_3_C_2_ multi-components nanocomposites for enhanced visible light photocatalytic hydrogen production. Chem. Phys. Lett..

[41] Ren Y.T., Dong T.A., Ding S.P., Liu X.F., Zheng H.Z., Gao L.L., Hu J.C. (2021). AgBr Nanoparticles Anchored on CdS Nanorods as Photocatalysts for H_2_ Evolution. ACS Appl. Nano Mater..

[42] Li P.X., Zhao H., Yan X.Y., Yang X., Li J.J., Gao S.Y., Cao R. (2020). Visible-light-driven photocatalytic hydrogen production coupled with selective oxidation of benzyl alcohol over CdS@MoS_2_ heterostructures. Sci. China-Mater..

[43] Li P.X., Yan X.Y., Gao S.Y., Cao R. (2021). Boosting photocatalytic hydrogen production coupled with benzyl alcohol oxidation over CdS/metal-organic framework composites. Chem. Eng. J..

[44] Jiang D.C., Chen X., Zhang Z., Zhang L., Wang Y., Sun Z.J., Irfan R.M., Du P.W. (2018). Highly efficient simultaneous hydrogen evolution and benzaldehyde production using cadmium sulfide nanorods decorated with small cobalt nanoparticles under visible light. J. Catal..

[45] Zhang L., Jiang D.C., Irfan R.M., Tang S., Chen X., Du P.W. (2019). Highly efficient and selective photocatalytic dehydrogenation of benzyl alcohol for simultaneous hydrogen and benzaldehyde production over Ni-decorated Zn_0.5_Cd_0.5_S solid solution. J. Energy Chem..

[46] Zhang F., Zhuang H.Q., Zhang W.M., Yin J., Cao F.H., Pan Y.X. (2019). Noble-metal-free CuS/CdS photocatalyst for efficient visible-light-driven photocatalytic H_2_ production from water. Catal. Today.

[47] Ahmad I., Shukrullah S., Naz M.Y., Bhatti H.N. (2023). A Cu medium designed Z-scheme ZnO-Cu-CdS heterojunction photocatalyst for stable and excellent H_2_ evolution, methylene blue degradation, and CO_2_ reduction. Dalton Trans..

[48] Jin Y.X., Zheng D.D., Fang Z.P., Pan Z.M., Wang S.B., Hou Y.D., Savateev O., Zhang Y.F., Zhang G.G. (2024). Salt-melt synthesis of poly(heptazine imide) in binary alkali metal bromides for enhanced visible-light photocatalytic hydrogen production. Interdiscip. Mater..

[49] Ren X., Shi J.Y., Duan R.H., Di J., Xue C., Luo X., Liu Q., Xia M.Y., Lin B., Tang W. (2022). Construction of high-efficiency CoS@Nb_2_O_5_ heterojunctions accelerating charge transfer for boosting photocatalytic hydrogen evolution. Chin. Chem. Lett..

[50] Grote F., Yu Z.Y., Wang J.L., Yu S.H., Lei Y. (2015). Self-Stacked Reduced Graphene Oxide Nanosheets Coated with Cobalt-Nickel Hydroxide by One-Step Electrochemical Deposition toward Flexible Electrochromic Supercapacitors. Small.

[51] Zhu C.X., Yang J.R., Zhang J.W., Wang X.Q., Gao Y., Wang D.S., Pan H.G. (2024). Single-atom materials: The application in energy conversion. Interdiscip. Mater..

[52] Chen G., Zhou Z., Li B., Lin X., Yang C., Fang Y., Lin W., Hou Y., Zhang G., Wang S. (2024). S-scheme heterojunction of crystalline carbon nitride nanosheets and ultrafine WO_3_ nanoparticles for photocatalytic CO_2_ reduction. J. Environ. Sci..

[53] Cai M., Liu Y., Dong K., Chen X., Li S. (2023). Floatable S-scheme Bi_2_WO_6_/C_3_N_4_/carbon fiber cloth composite photocatalyst for efficient water decontamination. Chin. J. Catal..

[54] Zhang P., Luan D., Lou X.W.D. (2020). Fabrication of CdS Frame-in-Cage Particles for Efficient Photocatalytic Hydrogen Generation under Visible-Light Irradiation. Adv. Mater..

