# Supplementary material for: Integrating Ni(OH)2 Nanoparticles on CdS for Efficient Noble-Metal-Free Photocatalytic H2 Evolution
Source: Molecules. 2024 Dec 10;29(24):5821. doi: 10.3390/molecules29245821 (PMC11677877; doi:10.3390/molecules29245821)
Supplement: Supplementary file 1 [file molecules-29-05821-s001.zip › molecules-3304556-supplementary.pdf]

Supporting Information

Integrating $Ni(OH)_2$ Nanoparticles on CdS for Efficient Noble-Metal-Free Photocatalytic H_2 Evolution

Zemeng Wang [1], Piaopiao Wu [1], Weiya Huang [1], Kai Yang [1], Kangqiang Lu [1,*] and Zhaoguo Hong [2,*]

1 Jiangxi Provincial Key Laboratory of Functional Crystalline Materials Chemistry, School of Chemistry and Chemical Engineering, Jiangxi University of Science and Technology, Ganzhou 341000, China; mmeng8686@163.com (Z.W.); 19142170804@163.com (P.W.); hweiya@126.com (W.H.); yangkai@jxust.edu.cn (K.Y.)

2 School of Pharmaceutical Sciences, Gannan Medical University, Ganzhou 341000, China

* Correspondence: kqlu@jxust.edu.cn (K.L.); 18677342951@163.com (Z.H.)

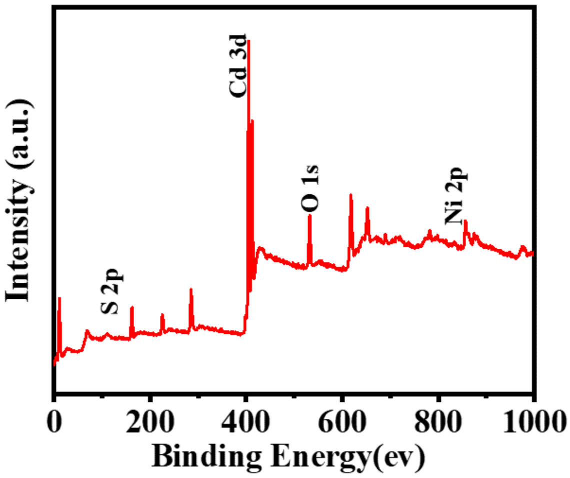

Figure S1. XPS spectra for the survey spectra of the CdS/5%$Ni(OH)_2$ composite.

Table S1 Surface Areas of Samples.

Samples	BET surface area ($m^2 \cdot g^{-1}$)	Pore Volume ($cm^3 \cdot g^{-1}$)	Pore Size (nm)
CdS	98.15	0.17	53.43
CdS/5%$Ni(OH)_2$	138.67	0.24	68.43
